# The Bio-Community Perl toolkit for microbial ecology

**DOI:** 10.1093/bioinformatics/btu130

**Published:** 2014-03-10

**Authors:** Florent E. Angly, Christopher J. Fields, Gene W. Tyson

**Affiliations:** ^1^Australian Centre for Ecogenomics, School of Chemistry and Molecular Biosciences, Level 5, Molecular Biosciences Building (76), The University of Queensland, Brisbane St Lucia, QLD 4072, Australia and ^2^HPCBio, Carver Biotechnology Center, Institute for Genomic Biology, 1206 West Gregory Drive | MC-195, Urbana, IL 61801, USA

## Abstract

**Summary:** The development of bioinformatic solutions for microbial ecology in Perl is limited by the lack of modules to represent and manipulate microbial community profiles from amplicon and meta-omics studies. Here we introduce Bio-Community, an open-source, collaborative toolkit that extends BioPerl. Bio-Community interfaces with commonly used programs using various file formats, including BIOM, and provides operations such as rarefaction and taxonomic summaries. Bio-Community will help bioinformaticians to quickly piece together custom analysis pipelines and develop novel software.

**Availability an implementation:** Bio-Community is cross-platform Perl code available from http://search.cpan.org/dist/Bio-Community under the Perl license. A readme file describes software installation and how to contribute.

**Contact:** f.angly@uq.edu.au

**Supplementary information:** Supplementary data are available at *Bioinformatics* online

## 1 INTRODUCTION

Sequencing is common in most fields of biological research, and the throughput of modern platforms is orders of magnitudes higher than traditional Sanger sequencing (Metzker, 2010). The BioPerl bioinformatic toolkit (Stajich *et al.*, 2002) has attracted a large community of users and developers and has become critical in many sequencing projects by allowing quick code development and interaction between programs using incompatible file formats. In microbial ecology, sequencing is used routinely for 16S rRNA gene amplicon surveys (Tringe and Hugenholtz, 2008), metagenomics (Handelsman, 2004) and metatranscriptomics (Frias-Lopez *et al.*, 2008). Because most microorganisms remain uncultivated (Rappé and Giovannoni, 2003), culture-independent molecular surveys are essential for the characterization of environmental microbial communities. However, they require large computational resources, novel bioinformatic tools and elaborate pipelines. Many tools have been developed to analyze the resulting sequence data. For example, libraries written in Python (Knight *et al.*, 2007) and R (Dixon, 2003; Kembel *et al.*, 2010) provide blocks for building bioinformatic software. QIIME (Caporaso *et al.*, 2010) and mothur (Schloss *et al.*, 2009) are dedicated packages with scripts to build complete analysis pipelines, but they use incompatible file formats. Here, we introduce Bio-Community, a set of format-agnostic modules and scripts to parse and manipulate taxonomic or functional microbial community profiles.

## 2 FEATURES

### 2.1 Object model

**Fig. 1.**
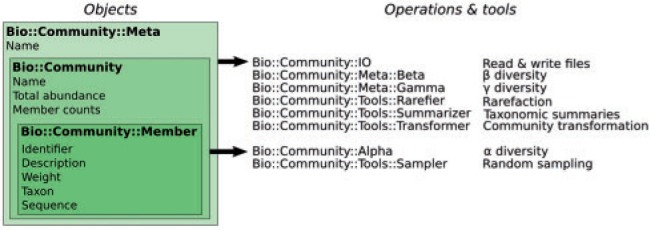
Main objects, their attributes and operation modules

Bio-Community is a Perl object-oriented toolkit that extends BioPerl. It is centered around the Community object, which contains a group of entities from the same geographic area (Fig. 1).

**Fig. 2.**
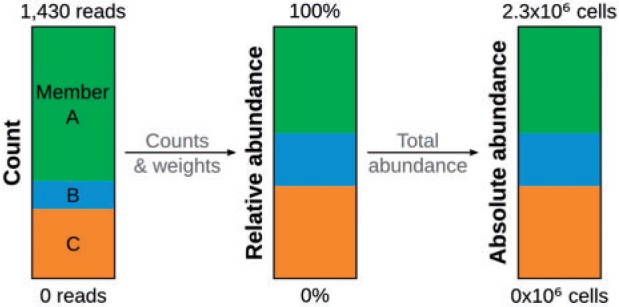
Relation between abundance types. Relative abundance depends on member counts and weights, whereas absolute abundance is further derived from a total abundance measure

These entities are Member objects, representing individual genomes, genes, taxa or operational taxonomic units from amplicon and meta-omic surveys. Member objects store attributes such as an identifier, a taxon or a sequence and can be given weights to account for the fact that there is no one-to-one relationship between a sequencing read and a microbial cell. The relative abundance or abundance rank of a Member can be calculated based on this Member’s count, weight and the total count in the Community (Fig. 2). Similarly, absolute abundance is based on total microbial abundance in the community, quantifiable by epifluorescence microscopy, qPCR or flow cytometry (Rinsoz *et al.*, 2008).

### 2.2 Diversity metrics

Bio-Community quantifies community α, β and γ diversity (Whittaker, 1972) using a range of metrics [reviewed by Magurran (2004)]. The diversity of a single Community object, α diversity, is represented by metrics of richness, evenness, dominance and indices (Supplementary Table S1). Several Community objects can be grouped into a Meta object, representing a metacommunity (Leibold *et al.*, 2004). This object provides methods to measure γ diversity, i.e. the collective diversity of its communities, and β diversity, i.e. their dissimilarity. The γ metrics are the same as those available for α diversity, whereas those for β diversity include qualitative and quantitative forms (Supplementary Table S1).

### 2.3 Data input and output

Community profiles (e.g. a site-by-species table) describe the distribution of members in biological samples. Operations to read and write these files are handled by the IO module and are important for exchanging data between programs using different formats. We have implemented parsers for five common file types (Supplementary Table S2), including the BIOM standard (McDonald *et al.*, 2012). Examples of these file types are given in the t/data folder of the Bio-Community package. The parsers automatically detect file format based on its content using the FormatGuesser module, and iteratively record member identifier, taxonomy and abundance.

### 2.4 Tools

**Fig. 3.**
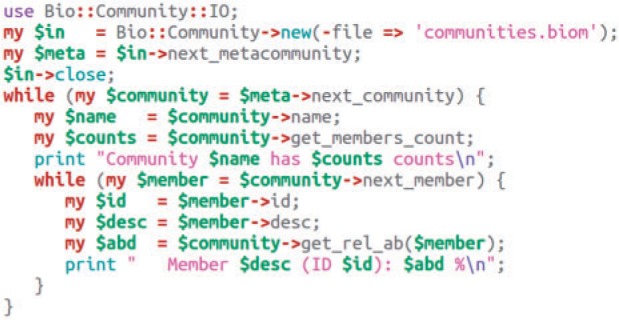
Vignette illustrating the use of Bio-Community to read a BIOM community profile and report member information

Tool modules can perform operations such as community transformation, rarefaction and taxonomic summaries (Fig. 1). Utility scripts using these modules are available in Bio-Community (Supplementary Table S3). They allow biologists to perform specific operations on community profiles, but they do not form an entire microbial analysis pipeline. These scripts can also be regarded as examples of integration of Bio-Community into bioinformatic scripts (Fig. 3). This integration can also leverage external modules to rapidly develop powerful custom scripts, e.g. Getopt::Euclid for handling command-line arguments, BioPerl modules for reading sequences or running external programs (e.g. BLAST) (Camacho *et al.*, 2009) and Statistics::R for using R libraries or visualization capabilities.

## 3 CONCLUSIONS

Bio-Community provides several file formats to interface with popular programs and will help bioinformaticians quickly construct custom analysis pipelines or novel software for microbial ecology. The integration of relative and absolute abundance with diversity metrics permits holistic microbial studies (Dinsdale *et al.*, 2008; Dove *et al.*, 2013; Nathani *et al.*, 2013), while weights can be added to account for gene copy number (Kembel *et al.*, 2012) or genome length (Angly *et al.*, 2009; Beszteri *et al.*, 2010) bias. We encourage programmers to join the development of Bio-Community at https://github.com/bioperl/Bio-Community and to add support for new file formats, diversity metrics or tools.

*Funding*: Australian Research Council DE120101213 to FEA and DP1093175 to GWT.

*Conflict of interest:* none declared.

## Supplementary Material

Supplementary Data
