# Supplementary material for: The Bio-Community Perl toolkit for microbial ecology
Source: Bioinformatics. 2014 Mar 10;30(13):1926–7. doi: 10.1093/bioinformatics/btu130 (PMC4071200; doi:10.1093/bioinformatics/btu130)
Supplement: Supplementary Data [file supp_30_13_1926__index.html]

The Bio-Community Perl toolkit for microbial ecology — The Bio-Community Perl toolkit for microbial ecology — The Bio-Community Perl toolkit for microbial ecology — Supplementary Data 

# The Bio-Community Perl toolkit for microbial ecology

## Supplementary Data

files

**Files in this Data Supplement:**

- Supplementary Data - pdf file
