# Supplementary material for: The Bio-Community Perl toolkit for microbial ecology
Source: Bioinformatics. 2014 Mar 10;30(13):1926–7. doi: 10.1093/bioinformatics/btu130 (PMC4071200; doi:10.1093/bioinformatics/btu130)
Supplement: Supplementary Data [file supp_btu130_Bio-Community_supplementary.pdf]

SUPPLEMENTARY MATERIAL

Supplementary Table S1. α and β diversity metrics implemented in Bio-Community.

Type	Subtype	Name
α	Richness	Observed, Menhinick (Menhinick, 1964), Margalef (Margalef, 1957), Chao 1 (Chao, 1984), ACE (Chazdon *et al.*, 1998)
α	Evenness	Buzas & Gibson (Buzas and Gibson, 1969), Heip (Heip, 1974), Shannon (Shannon and Weaver, 1963; Pielou, 1975), Simpson (Simpson, 1949; Pielou, 1975), Brillouin (Brillouin, 1962; Pielou, 1975), Hill $E_{2,1}$ (Hill, 1973), McIntosh (McIntosh, 1967; Pielou, 1975), Camargo (Camargo, 1993)
α	Dominance	Simpson (Simpson, 1949), Berger-Parker (Berger and Parker, 1970)
α	Index (composite)	Shannon-Wiener (Shannon and Weaver, 1963), Simpson 1-D & 1/D (Simpson, 1949), Brillouin (Brillouin, 1962), Hill N_{inf} (Hill, 1973), McIntosh (McIntosh, 1967)
β	Qualitative (presence/absence)	Jaccard (Jaccard, 1901) and Sørensen (Sørensen, 1948; Whittaker, 1972)
β	Quantitative	1-norm (Manhattan distance), 2-norm (euclidean distance), infinity norm, Hellinger (Hellinger, 1909), Bray-Curtis (Bray and Curtis, 1957), Morisita-Horn (Horn, 1966) and MaxiPhi (Angly *et al.*, 2006)

Supplementary Table S2. Bio-Community supported input and output file formats.

Name	Format description	Number of samples	Member information
generic	Tab-delimited, site-by-species	Multiple	Abundance and description
qiime	Tab-delimited QIIME (Caporaso *et al.*, 2010)	Multiple	Abundance, ID and optional description
gaas	Tab-delimited GAAS (Angly *et al.*, 2009)	Single	Abundance, ID and description
unifrac	Tab-delimited UniFrac (Lozupone *et al.*, 2006)	Multiple	Abundance and description
biom	JSON-based format (McDonald *et al.*, 2012)	Multiple	Abundance, ID, description and other metadata

Supplementary Table S3. Utility scripts included in Bio-Community.

Name	Description
bc_clean_shrapnel	Remove low-count, low-prevalence community members
bc_convert_files	Merge or split community files and convert between different formats
bc_correct_misassignments	Try fixing incorrect taxonomic assignments, using a reference community
bc_get_info	Get information about a community profile (samples, members or file format)
bc_manage_samples	Include, delete, merge, sort or rename samples
bc_measure_alpha	Measure α diversity of communities
bc_measure_beta	Measure β diversity between communities
bc_rarefy	Rarefy communities
bc_relative_to_absolute	Convert relative abundances into absolute abundance
bc_remove_unexpected_members	Remove members not expected to occur, based on a reference community
bc_summarize	Summarize community composition at different taxonomic levels
bc_use_repr_id	Replace member ID by that of their OTU cluster or taxonomic representative

REFERENCES

Angly,F.E. *et al.* (2009) The GAAS metagenomic tool and its estimations of viral and microbial average genome size in four major biomes. *PLoS Comput. Biol.*, **5**, e1000593.

Angly,F.E. *et al.* (2006) The marine viromes of four oceanic regions. *PLoS Biol.*, **4**, e368.

Berger,W.H. and Parker,F.L. (1970) Diversity of planktonic foraminifera in deep-sea sediments. *Science*, **168**, 1345–134.

Bray,J.R. and Curtis,J.T. (1957) An ordination of the upland forest communities of Southern Wisconsin. *Ecol. Monogr.*, **27**, 326–349.

Brillouin,L. (1962) Science and information theory Academic Press, New York.

Buzas,M.A. and Gibson,T.G. (1969) Species diversity: benthonic Foraminifera in western North Atlantic. *Science*, **163**, 72–75.

Camargo,J.A. (1993) Must dominance increase with the number of subordinate species in competitive interactions ? *J. Theor. Biol.*, **161**, 537–542.

Caporaso,J.G. *et al.* (2010) QIIME allows analysis of high-throughput community sequencing data. *Nat. Methods*, **7**, 335–336.

Chao,A. (1984) Nonparametric estimation of the number of classes in a population. *Scand. J. Stat.*, **11**, 265–270.

Chazdon,R.L. *et al.* (1998) Statistical methods for estimating species richness of woody regeneration in primary and secondary rain forests of Northeastern Costa Rica.

Heip,C. (1974) A new index measuring evenness. *J. Mar. Biol. Assoc. U. K.*, **54**, 555–557.

Hellinger,E. (1909) Neue Begründung der Theorie quadratischer Formen von unendlich vielen Veränderlichen. *J. Für Reine Angew. Math.*, **136**, 210–271.

Hill,M.O. (1973) Diversity and evenness: a unifying notation and its consequences. *Ecology*, **54**, 427–432.

Horn,H.S. (1966) Measurement of ‘overlap’ in comparative ecological studies. *Am. Nat.*, **100**, 419–424.

Jaccard,P. (1901) Étude comparative de la distribution florale dans une portion des Alpes et des Jura. *Bull. Société Vaudoise Sci. Nat.*, **37**, 547–579.

Lozupone,C. *et al.* (2006) UniFrac - An online tool for comparing microbial community diversity in a phylogenetic context. *BMC Bioinformatics*, **7**, 371.

Margalef,R. (1957) The theory of information in ecology. *Gen. Syst. Bull.*, **3**, 36–71.

McDonald,D. *et al.* (2012) The Biological Observation Matrix (BIOM) format or: how I learned to stop worrying and love the ome-ome. *GigaScience*, **1**, 7.

McIntosh,R.P. (1967) An index of diversity and the relation of certain concepts to diversity. *Ecology*, **48**, 392.

Menhinick,E.F. (1964) A comparison of some species-individuals diversity indices applied to samples of field insects. *Ecology*, **45**, 859.

Pielou,E.C. (1975) Ecological diversity Wiley, New York.

Shannon,C.E. and Weaver,W. (1963) The mathematical theory of communication Univ of Illinois Press, Urbana.

Simpson,E.H. (1949) Measurement of diversity. *Nature*, **163**, 688.

Sørensen,T.A. (1948) A method of establishing groups of equal amplitude in plant sociology based on similarity of species content, and its application to analyses of the vegetation on Danish commons. *K. Dan. Vidensk. Selsk. Biol. Skr.*, **5**, 1–34.

Whittaker,R.H. (1972) Evolution and measurement of species diversity. *Taxon*, **21**, 213–251.
